# Adherence to the Swedish dietary guidelines and the impact on mortality and climate in a population-based cohort study

**DOI:** 10.1017/S1368980023001295

**Published:** 2023-11

**Authors:** Anna Strid, Elinor Hallström, Anna Karin Lindroos, Bernt Lindahl, Ingegerd Johansson, Anna Winkvist

**Affiliations:** 1 Department of Internal Medicine and Clinical Nutrition, The Sahlgrenska Academy, University of Gothenburg, SE-405 30 Gothenburg, Sweden; 2 Department of Agriculture and Food, Research Institutes of Sweden (RISE), Lund, Sweden; 3 Department of Risk and Benefit Assessment, Swedish Food Agency, Uppsala, Sweden; 4 Sustainable Health, Department of Public Health and Clinical Medicine, Umeå University, Umeå, Sweden; 5 Department of Odontology, Umeå University, Umeå, Sweden

**Keywords:** Food-based dietary guidelines, Diet quality, Dietary indices, Sustainability, Sustainable diets

## Abstract

**Objective:**

To assess the associations between adherence to the Swedish dietary guidelines and all-cause mortality (i.e. assessing the index’ ability to predict health outcomes), as well as levels of dietary greenhouse gas emissions (GHGEs).

**Design:**

A longitudinal study 1990–2016 within the population-based cohort Västerbotten Intervention Programme. Dietary data were based on FFQs. Diet quality was assessed by the Swedish Healthy Eating Index for Adults 2015 (SHEIA15), based on the 2015 Swedish dietary guidelines. Dietary GHGEs were estimated from life cycle assessment data including emissions from farm to industry gate. Hazard ratios (HR) and 95 % CI of all-cause mortality were evaluated with Cox proportional hazards regression, and differences in median GHGEs were tested between quintiles of SHEIA15 score using the Kruskal–Wallis one-way ANOVA test.

**Setting:**

Northern Sweden.

**Participants:**

In total, 49 124 women and 47 651 men, aged 35–65 years.

**Results:**

Median follow-up times were 16·0 years for women and 14·7 years for men, during which time 3074 women and 4212 men died. A consistent trend of lower all-cause mortality HR for both sexes with higher SHEIA15 scores was demonstrated. For women, the all-cause mortality HR was 0·81 ((95 % CI 0·71, 0·92); *P* = 0·001) and for men 0·90 ((95 % CI 0·81, 0·996); *P* = 0·041) between the quintile with the highest SHEIA15 score compared with the quintile with the lowest SHEIA15 score. A consistent trend of lower estimated dietary GHGEs among both sexes with higher SHEIA15 scores was also found.

**Conclusions:**

Adherence to Swedish dietary guidelines, estimated by SHEIA15, seems to promote longevity and reduce dietary climate impact.

A large part of the noncommunicable disease burden in the world is related to poor dietary habits^(1)^. This implies that improvements in public health through lifestyle changes are possible and necessary. To aid the prevention of chronic diseases and the promotion of public health by healthy diets, over 100 countries in the world have developed national food-based dietary guidelines (FBDGs)^(2)^. These FBDGs build on existing scientific evidence with an adaptation to the dietary habits, nutritional status, food culture and food availability of the specific country^(2)^. Several countries have also recognised the opportunity and responsibility to incorporate further aspects of sustainability in their FBDGs^(3)^, and in 2019, FAO and WHO published guiding principles to support and encourage this undertaking^(4)^. One of the first countries integrating environmental aspects in their FBDGs, in addition to nutritional and public health aspects, was Sweden in 2015^(5)^. This was facilitated by the Swedish Food Agency, publisher of the Swedish FBDGs, having responsibility of both public health and environmental objectives.

To enable assessments of adherence to the FBDGs in a specific population, indices assessing different aspects of dietary intake are proposed as useful tools in epidemiologic research^(6,7)^. However, a large number of diet quality indices exist in the literature, and the choices made in regard to the development of these indices, such as food items or nutrients included, cut-off values used and scoring method have been deemed subjective and arbitrary^(8)^. Hence, these diet quality indices need to be validated^(7)^.

To assess the adherence to the 2015 Swedish FBDGs, including both health and environmental aspects, Moraeus et al. developed a diet quality index in 2020^(9)^. However, the suggested diet quality index has not yet been validated to assure the ability to predict health and environmental outcomes. Hence, there were two objectives of the current study. The first objective was to assess the association between the proposed diet quality index developed to estimate adherence to the 2015 Swedish FBDGs, the Swedish Healthy Eating Index for Adults 2015 (SHEIA15), and all-cause mortality, thus assessing the index’ ability to predict health outcomes. The second objective was to investigate the differences in greenhouse gas emissions (GHGEs) from diets of participants with increasing adherence to the 2015 Swedish FBDGs, according to SHEIA15, to learn if there are possible gains in climate of following the 2015 Swedish FBDGs.

## Subjects and methods

### Study design and study participants

**Fig. 1:**
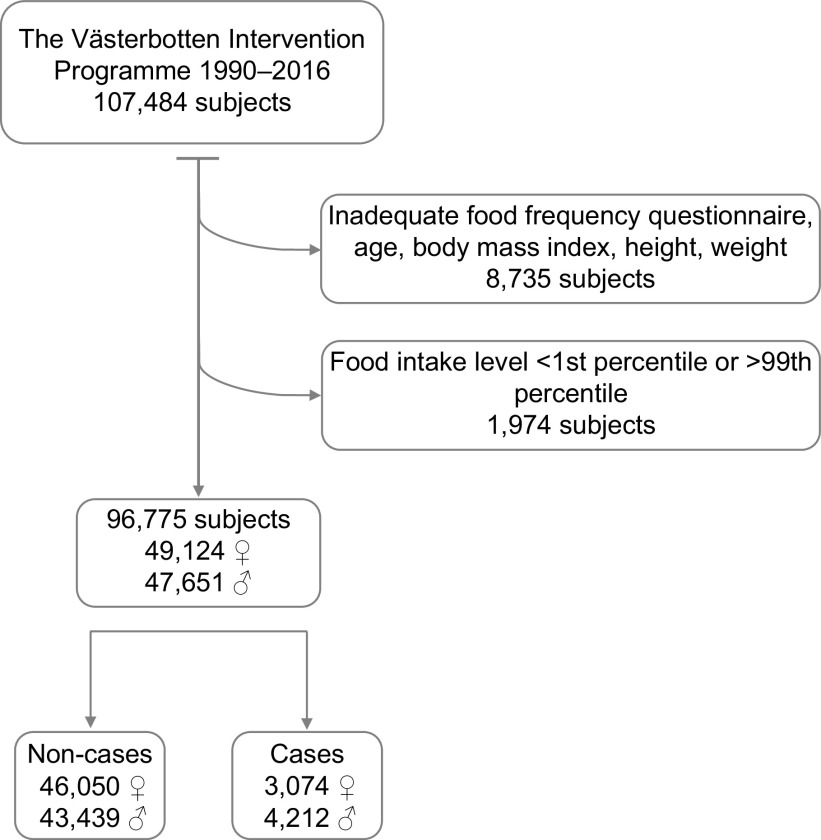
Flow chart from inclusion of participants in the Västerbotten Intervention Programme with their baseline study visit 1990–2016, to the final study group in the current study. Participants in the final study group in the current study are presented as non-cases and cases of all-cause mortality separated by gender.

The Västerbotten Intervention Programme (VIP) is a population-based prospective cohort study in northern Sweden, where inhabitants in Västerbotten are invited to their regular health care centre for health check-ups^(10)^. The baseline study visits include standardised assessments of anthropometrics, measurement of blood pressure (after a 5-min rest), drawing of blood samples (after a ≥ 4-h fast) and an extensive questionnaire on diet, lifestyle and socio-economic factors. More information on the study design of VIP can be found in Norberg et al.^(10)^. For the present study, VIP participants undergoing their baseline visit between 1990 and 2016 were included. During that time period, 107 484 subjects contributed to 158 484 observations. Only one observation per subject was included in the analyses. To ensure reliable data, the following exclusion criteria were employed: (i) if the reporting of food intake was incomplete (≥ 10 % missing intake data and/or missing portion size recording); (ii) a height < 130 cm or > 210 cm; (iii) a body weight < 35 kg; (iv) a BMI < 15 kg/m^2^ or data missing; (v) age < 35 or > 65 years and (vi) food intake levels < 1st or > 99th percentiles^(11)^. Figure 1 presents a flow chart from inclusion of participants in VIP during the period of 1990–2016, to the final study group in the current study.

### Dietary assessment

Self-reported dietary data were provided by 49 124 women and 47 651 men on their baseline visit using a semi-quantitative retrospective FFQ that referred to the intake of both single food items and food groups the previous twelve months. During the study period (1990–2016), two versions of the FFQ have been used. Before 1996, eighty-four questions on food intake were included, and after 1996, the FFQ was reduced to sixty-four questions by merging similar foods from the earlier version and removing more uncommon foods^(12)^. Approximately 28 % of the participants in the current study answered the longer FFQ version and 72 % answered the shorter version. Intakes reported by the two different FFQs have been harmonised. Estimation of portion sizes was either aided by four pictures of increasing portion sizes of protein sources (fish and/or meat), staple foods (pasta, rice and/or potato) and vegetables, or by fixed sizes (e.g. fruit), or age and gender-specific portion sizes^(10)^. Ten repeated 24-h recalls in 195 of the study participants was performed to validate the longer FFQ version^(13)^.

### Estimation of nutrient density of diets

Daily energy intake and nutrient intake levels were estimated with data from the national food composition database at the Swedish Food Agency (https://soknaringsinnehall.livsmedelsverket.se/). The reported food intake referred to prepared food when relevant. Estimation of intake of added sugars was based on unpublished data informed by the Swedish Food Agency, calculated according to a method comprising ten steps by Wanselius et al.^(14)^. Energy adjustment of nutrient intakes was performed to 2000 kcal per day for women and 2500 kcal per day for men, to correct for expected underreporting of dietary intake and to enable assessment of quality of the diet rather than quantity of the diet. We have previously estimated the underreporting among the VIP participants to be 47 % for women and 44 % for men, respectively, as judged by a food intake level (reported energy intake divided by estimated BMR) below 1·2^(15)^.

Diet quality was assessed by SHEIA15, which was originally developed for adolescents by Moraeus et al.^(9)^, with calculations adapted from Knudsen et al.^(16)^. SHEIA15 is based on the Nordic Nutrition Recommendations from 2012 (NNR 2012) and the most recent Swedish dietary guidelines from 2015^(5,17)^. SHEIA15 includes nine components, of which six are positive components (vegetables and fruit, seafood, whole grains, dietary fibre, MUFA, and PUFA) and three are negative components (SFA, red and processed meat, and added sugar). SHEIA15 is built by calculating the ratio of the reported intake and the recommended intake of each component. Recommended intakes for women and men and information on the calculation of each component are displayed in Table 1. By calculating ratios, all intakes are considered. Ratio values above 1 and below 0 are recoded to 1 and 0. The scores of the individual components are summed to a total score with a minimum of zero and a maximum of nine, where nine indicates the highest adherence to the 2015 Swedish FBDGs.

**Table 1:** Components of the Swedish Healthy Eating Index for Adults 2015 (SHEIA15), associated recommended intakes and calculation of the SHEIA15 score

Components	Recommended intakes	Calculation of SHEIA15
**Positive components**		
Vegetables and fruit	> 500 g/d*	Reported intake in g/500
Dietary fibre	25–35 g/d^†^	Reported intake in g/25 or 35
Wholegrains	> 75 g/10MJ^§^	Reported intake in g/68 or 78
Seafood	45 g/d*,**	Reported intake in g/45
PUFA	> 7·5 E%^†^	E%/7·5
MUFA	> 15 E%^†^	E%/15
**Negative components**		
SFA	< 10 E%^†^	1 – ((E% – 10)/10)
Red and processed meat	< 500 g/week*	1 – ((Reported intake in g – 500)/500)
Added sugar	< 10 E%^†^	1 – ((E% – 10)/10)

E%, percent of total energy.

The table is adapted from Moraeus et al.^(9)^

* Based on the Swedish food-based dietary guidelines from 2015.

† Based on the Nordic Nutrition Recommendations 2012.

§ Based on Becker W, Busk L, Mattisson I et al. (2012) Råd om fullkorn 2009 – bakgrund och vetenskapligt underlag. (Guidelines about wholegrains 2009 – background and scientific basis) (in Swedish). NFA report no. 102 012. Uppsala.

** Based on 2–3 times per week with a portion size of 125 g.

### Estimation of dietary GHGE

A more detailed description of the estimation of dietary GHGEs of all participants in VIP during the years 1990–2016 can be found elsewhere^(18)^. In brief, life cycle assessment data from the Research Institutes of Sweden Food and Climate Database was used to estimate GHGEs. GHGEs were expressed as kg carbon dioxide equivalents (CO_2_e) per kg edible food product^(19,20)^ and referred to prepared food when relevant. GHGEs included were from farm to industry gate. The daily dietary GHGEs was adjusted for energy intake for all study participants and is expressed as kg CO_2_e per 2000 kcal for women and kg CO_2_e per 2500 kcal for men.

### Non-dietary variables

The baseline standardised health screening and the questionnaire on lifestyle and socio-economic factors provided all information on non-dietary variables, and a more extensive description can be found in Norberg et al.^(10)^. Importantly, the weight and height of the participants were measured in standardised methods, and BMI was calculated (kg/m^2^). BMI was further classified into underweight (< 18·5), normal weight (18·5–25·0), overweight (> 25·0–30·0) and obese (> 30·0). The validated Cambridge Index of Physical Activity was used to assess physical activity^(21)^, which was separated into (i) inactive, (ii) moderately inactive, (iii) moderately active and (iv) active, based on both working hours and leisure time. Level of education was separated into three categories: (i) basic level of 9 years of school; (ii) high school and (iii) university. Smoking status was separated into three categories; (i) currently smoking; (ii) have smoked and (iii) not smoking.

### Assessment of all-cause mortality

All-cause mortality data was attained by linking the VIP participants using personal identification numbers to the ‘Cause of death’ registers at the National Board of Health and Welfare in Sweden (https://www.socialstyrelsen.se/statistik-och-data/register/). Here, all causes of death in Sweden are included.

### Data processing and statistical analyses

The participants were separated into quintiles of SHEIA15 score, ranked within age groups at baseline (35–44 years, 45–54 years and 55–65 years) within each gender. Quintile one had the lowest SHEIA15 score, and hence the lowest adherence to the 2015 Swedish FBDGs, and quintile five had the highest SHEIA15 score, and hence the highest adherence to the 2015 Swedish FBDGs. All analyses were stratified on gender.

Multivariable Cox proportional hazards regression was used to evaluate the association between adherence to the 2015 Swedish FBDGs, assessed by SHEIA15, and all-cause mortality. Hazard ratios (HR) and 95 % CI of all-cause mortality between the reference group quintile one and quintiles two to five were estimated. The follow-up time, i.e. the months between the baseline study visit (1990–2016) and death (cases) or end of the study period (31 December 2016) (non-cases), was used as the measurement of time in the Cox analyses. Age and age squared were included as potential confounders in the basic model. The purposeful selection method for model building^(22)^, also called the Bursac method, was performed to test for potential confounders and thus to select covariates for the multivariable Cox regression analysis. All covariates that were indicated as potential confounders by the Bursac method overall for either women or men were included in the final adjusted model. Hence, in the adjusted models, age, age squared, BMI, educational level, physical activity, smoking status and year of study participation were included as potential confounders. The proportional hazards assumption was assured for the fully adjusted model.

To evaluate the differences in dietary GHGEs of participants with increasing adherence to the 2015 Swedish FBDGs, i.e. increasing SHEIA15 score, the non-parametric Kruskal–Wallis one-way ANOVA test was performed on the quintiles of SHEIA15. Pairwise comparisons of dietary GHGEs between all quintiles were tested with the Dunn post hoc test, and the significance values were adjusted by the Bonferroni correction for multiple tests. The non-parametric Jonckheere Trend test was further performed to test for a trend between the SHEIA15 quintiles and dietary GHGEs.

Sensitivity analyses were performed to assess whether the associations seen in the Cox analyses and the Kruskal–Wallis one-way ANOVA tests were driven by a single component of the SHEIA15 score. This was done by excluding the components of the SHEIA15 one at a time. The Cox analyses were performed on the continuous scale of SHEIA15 and analyses of median GHGE were performed between quintile one and quintile five with the Mann–Whitney U test.

Descriptive statistics were used to define differences in baseline characteristics between non-cases and all-cause mortality cases (Table 2), as well as between the quintiles of SHEIA15 score (online Supplementary Tables 1–2). Descriptive statistics were also used to define differences between the quintiles of SHEIA15 score in the reported food intakes and estimated nutrient intake of the nine components of the SHEIA15 (online Supplementary Tables 3–4).

**Table 2:** Baseline characteristics of participating women and men (*n* 96 775) in the population-based prospective cohort Västerbotten Intervention Programme between 1990 and 2016, separated by non-cases and all-cause mortality cases during follow-up

	Women (*n* 49 124)				Men (*n* 47 651)			
	Non-cases (*n* 46 050)		Cases (*n* 3074)		Non-cases (*n* 43 439)		Cases (*n* 4212)	
	Mean or %	sd	Mean or %	sd	Mean or %	sd	Mean or %	sd
Age* (years)	47·4	7·9	54·1	6·8	47·4	7·8	54·2	6·7
BMI* (kg/m^2^)	25·6	4·6	26·1	5·0	26·5	3·8	27·0	3·9
Underweight, < 18·5 (%)	1·0	–	1·6	–	0·3	–	0·5	–
Normal, 18·5–25·0 (%)	52·2	–	43·0	–	36·7	–	33·9	–
Overweight, > 25·0–30·0 (%)	31·5	–	34·9	–	47·9	–	48·5	–
Obese, > 30·0 (%)	15·2	–	20·4	–	15·1	–	17·2	–
Physical activity (%)								
Inactive	17·4	–	21·5	–	18·2	–	21·2	–
Moderately inactive	30·3	–	35·9	–	29·7	–	35·7	–
Moderately active	27·8	–	26·5	–	28·8	–	27·9	–
Active	24·2	–	14·8	–	23·0	–	14·4	–
Missing value	0·4	–	1·2	–	0·3	–	0·9	–
Level of education (%)								
Basic level, 9 years	33·2	–	70·0	–	37·0	–	70·1	–
High school	30·1	–	13·1	–	35·7	–	15·0	–
University	36·1	–	15·3	–	26·8	–	13·8	–
Missing value	0·7	–	1·6	–	0·5	–	1·1	–
Smoking (%)								
Currently smoking	19·7	–	33·5	–	17·6	–	31·2	–
Have smoked	29·0	–	24·4	–	30·7	–	34·6	–
Do not smoke	50·4	–	41·0	–	50·3	–	32·5	–
Missing value	0·9	–	1·1	–	1·5	–	1·7	–

The table is adapted from Strid et al^(15)^.

* Adjusted for age and year of study participation.

For statistical analyses, SPSS version 28.0.1.1 (IBM SPSS Statistics) was used. The significance level was set at *P* < 0·05, unless otherwise stated. The median value and the first and third quartiles values are used to present non-normally distributed variables, and the mean value and sd are used to present normally distributed variables.

## Results

### Study participants

During 1990–2016, 54 620 women and 52 864 men participated in VIP, and of those participants 49 124 women and 47 651 men were included in the current study (Fig. 1). Median follow-up times were 16·0 years for women and 14·7 years for men, during which time 3074 women and 4212 men died, respectively. Approximately 36 % of the women and 27 % of the men reported an educational level up to and including university among the non-cases, and the corresponding figures for the cases were 15 % for the women and 14 % for the men. Further, almost 20 % of the women and 18 % of the men among the non-cases reported that they were currently smoking at baseline, and the corresponding figures for the cases were 34 % for the women and 31 % for the men (Table 2).

### SHEIA15 and all-cause mortality

Statistically significantly lower all-cause mortality hazards were found for women in all quintiles of SHEIA15 score compared with quintile one, with a range from the highest quintile (HR 0·81 (95 % CI 0·71, 0·91); *P* = 0·001) to the second lowest quintile (HR 0·86 (95 % CI 0·78, 0·95); *P* = 0·004) (Table 3). Statistically significantly lower all-cause mortality hazards were also found for men in quintiles five (HR 0·90 (95 % CI 0·81, 0·996); *P* = 0·041), four (HR 0·88 (95 % CI 0·80, 0·97); *P* = 0·009) and two (HR 0·90 (95 % CI 0·82, 0·98); *P* = 0·019) of SHEIA15 score, compared with quintile one (Table 3). In sensitivity analyses where the components of SHEIA15 were excluded one at a time, the results were robust, indicating that no single component of SHEIA15 was more important than the others (Table 4).

**Table 3:** Hazard ratios (HR) and 95 % CI for all-cause mortality of women (*n* 49 124) and men (*n* 47 651) classified into quintiles according to their diet quality estimated by SHEIA15*

	Quintiles of SHEIA15, women												
	Quintile 5 (*n* total 9824, *n* cases 377)			Quintile 4 (*n* total 9826, *n* cases 510)			Quintile 3 (*n* total 9825, *n* cases 599)			Quintile 2 (*n* total 9826, *n* cases 713)			Quintile 1 Reference group (*n* total 9823, *n* cases 875)
	HR	95 % CI	*P*	HR	95 % CI	*P*	HR	95 % CI	*P*	HR	95 % CI	*P*	HR
HR^†^ (95 % CI) basic model	0·69	0·61, 0·78	< 0·001	0·72	0·64, 0·80	< 0·001	0·73	0·66, 0·81	< 0·001	0·80	0·73, 0·89	< 0·001	1·00
HR^‡^ (95 % CI) adjusted model	0·81	0·71, 0·92	0·001	0·82	0·73, 0·92	< 0·001	0·84	0·75, 0·93	0·001	0·86	0·78, 0·95	0·004	1·00

	Quintiles of SHEIA15, men												
	Quintile 5 (*n* total 9529, *n* cases 610)			Quintile 4 (*n* total 9531, *n* cases 761)			Quintile 3 (*n* total 9531, *n* cases 929)			Quintile 2 (*n* total 9531, *n* cases 907)			Quintile 1 Reference group (*n* total 9529, *n* cases 1005)
	HR	95 % CI	*P*	HR	95 % CI	*P*	HR	95 % CI	*P*	HR	95 % CI	*P*	HR
HR^†^ (95 % CI) basic model	0·73	0·66, 0·81	< 0·001	0·77	0·70, 0·84	< 0·001	0·87	0·79, 0·95	0·002	0·85	0·77, 0·93	< 0·001	1·00
HR^‡^ (95 % CI) adjusted model	0·90	0·81, 0·996	0·041	0·88	0·80, 0·97	0·009	0·95	0·86, 1·04	0·244	0·90	0·82, 0·98	0·019	1·00

* The women and men were participants in the population-based Västerbotten Intervention Programme during the period 1990–2016. HR, 95 % CI and *P* values estimated by the Cox proportional hazards regression. Diet quality is estimated by the Swedish Healthy Eating Index for Adults (SHEIA15), which is based on the adherence to the Swedish dietary guidelines from 2015. Ranking into quintiles was adjusted by age groups (35–44 years, 45–54 years and 55–65 years). Quintile 1 is the lowest quintile and Quintile 5 the highest quintile. The SHEIA15 score has been energy adjusted to 2000 kcal for women and 2500 kcal for men.

† Basic model is adjusted for age and age squared.

‡ Adjusted model is adjusted for age, age squared, BMI, physical activity, educational level, smoking status and year of participation.

**Table 4:** Hazard ratios (HR) and 95 % CI for all-cause mortality of women and men with their diet quality estimated by SHEIA15 and the effect of the exclusion of components from the index*

	Women (*n* 49 124)			Men (*n* 47 651)		
	HR	95 % CI	*P*	HR	95 % CI	*P*
SHEIA15 with all nine components	0·91	0·87, 0·95	< 0·001	0·96	0·92, 0·99	0·016
SHEIA15 without vegetables and fruit	0·91	0·87, 0·97	0·001	0·95	0·91, 0·99	0·018
SHEIA15 without fibre	0·90	0·85, 0·95	< 0·001	0·95	0·91, 0·99	0·021
SHEIA15 without wholegrains	0·90	0·86, 0·95	< 0·001	0·96	0·92, 1·00	0·074
SHEIA15 without seafood	0·89	0·84, 0·93	< 0·001	0·95	0·91, 0·99	0·008
SHEIA15 without PUFA	0·89	0·85, 0·94	< 0·001	0·96	0·92, 0·99	0·016
SHEIA15 without MUFA	0·91	0·87, 0·95	< 0·001	0·96	0·93, 0·99	0·014
SHEIA15 without SFA	0·90	0·85, 0·95	< 0·001	0·96	0·92, 0·995	0·029
SHEIA15 without added sugar	0·91	0·86, 0·95	< 0·001	0·96	0·92, 0·995	0·026
SHEIA15 without red and processed meat	0·92	0·87, 0·96	< 0·001	0·95	0·92, 0·99	0·014

* The women and men were participants in the population-based Västerbotten Intervention Programme during the period 1990–2016. HR, 95 % CI and *P* values estimated by the Cox proportional hazards regression. Diet quality is estimated by the Swedish Healthy Eating Index for Adults (SHEIA15), which is based on the adherence to the Swedish dietary guidelines from 2015 and includes nine components. The SHEIA15 score has been energy adjusted to 2000 kcal for women and 2500 kcal for men. The model is adjusted for age, age squared, BMI, physical activity, educational level, smoking status and year of participation.

### SHEIA15 and GHGEs

Statistically significantly lower estimated dietary GHGEs were found for both women and men with an increasing SHEIA15 score (*P* < 0·001); medians ranging between 2·9–3·2 kg CO_2_e/2000 kcal and day for women and 3·4–4·1 kg CO_2_e/2500 kcal and day for men. All pairwise comparisons of differences in dietary GHGEs between all quintiles were also highly significant (*P* < 0·001), except for quintile four compared with quintile five for the women (*P* = 0·061) (Table 5). The trend tests between the SHEIA15 quintiles and dietary GHGEs were also highly significant (*P* < 0·001). In sensitivity analyses where the components of the SHEIA15 were excluded one at a time, analyses of differences in median GHGEs between quintile one and quintile five of SHEIA15 score were shown to be robust, except for when the component red and processed meat was excluded from the index (Table 6). Here, the median GHGEs for quintile one was slightly higher and the median GHGEs for quintile five was lower when red and processed meat was excluded from the index compared to when included, especially for the men where differences between quintile one and quintile five in dietary GHGEs now were non-significant (*P* = 0·073) (Table 6).

**Table 5:** Median dietary GHGEs for women (*n* 49 124) and men (*n* 47 651) classified into quintiles according to their diet quality estimated by SHEIA15*

	Quintiles of SHEIA15, women										
	Quintile 5 (*n* 9824)		Quintile 4 (*n* 9826)		Quintile 3 (*n* 9825)		Quintile 2 (*n* 9826)		Quintile 1 (*n* 9823)		
	Median	1st; 3rd quartile	Median	1st; 3rd quartile	Median	1st; 3rd quartile	Median	1st; 3rd quartile	Median	1st; 3rd quartile	*P* ^†^
GHGEs per day and 2000 kcal, kg CO_2_e	2·9	2·5; 3·3	2·9	2·5; 3·4	3·0	2·5; 3·5	3·0	2·6; 3·6	3·2	2·7; 3·9	< 0·001

	Quintiles of SHEIA15, men										
	Quintile 5 (*n* 9529)		Quintile 4 (*n* 9531)		Quintile 3 (*n* 9531)		Quintile 2 (*n* 9531)		Quintile 1 (*n* 9529)		
	Median	1st; 3rd quartile	Median	1st; 3rd quartile	Median	1st; 3rd quartile	Median	1st; 3rd quartile	Median	1st; 3rd quartile	*P* ^†^
GHGEs per day and 2500 kcal, kg CO_2_e	3·4	2·9; 3·9	3·6	3·1; 4·1	3·7	3·1; 4·3	3·8	3·2; 4·6	4·1	3·4; 5·0	< 0·001

Abbreviations: GHGEs, greenhouse gas emissions; CO_2_e, carbon dioxide equivalents.

* The women and men were participants in the population-based Västerbotten Intervention Programme during the period 1990–2016. Values are medians and 1st; 3rd quartiles. Diet quality is estimated by the Swedish Healthy Eating Index for Adults 2015 (SHEIA15), which is based on the adherence to the Swedish dietary guidelines from 2015. Ranking into quintiles was adjusted by age groups (35–44 years, 45–54 years and 55–65 years). Quintile 1 is the lowest quintile and Quintile 5 the highest quintile. The dietary GHGEs include emissions from farm to industry gate and have been energy adjusted to 2000 kcal for women and 2500 kcal for men.

† Differences between quintiles were tested using the non-parametric Kruskal–Wallis one-way ANOVA test. Pairwise comparisons of differences in GHGEs between all quintiles were *P* < 0·001, except for quintile 3 compared with quintile 4 for the women (*P* = 0·061), tested with the Dunn post hoc test with significance values adjusted by the Bonferroni correction for multiple tests. The trend between the SHEIA15 quintiles and dietary GHGEs was *P* < 0·001, tested with the non-parametric Jonckheere Trend test.

**Table 6:** Median dietary GHGEs for women and men classified into quintiles according to their diet quality estimated by SHEIA15 and the effect of the exclusion of components from the index^*^

	Women (*n* 49 124)					Men (*n* 47 651)				
	SHEIA15 Quintile 5 (*n* 9824)		SHEIA15 Quintile 1 (*n* 9823)			SHEIA15 Quintile 5 (*n* 9529)		SHEIA15 Quintile 1 (*n* 9529)		
	GHGEs per day and 2000 kcal, kg CO_2_e					GHGEs per day and 2500 kcal, kg CO_2_e				
	Median	1st; 3rd quartile	Median	1st; 3rd quartile	*P* ^†^	Median	1st; 3rd quartile	Median	1st; 3rd quartile	*P* ^†^
SHEIA15 with all nine components	2·9	2·5; 3·3	3·2	2·7; 3·9	< 0·001	3·4	2·9; 3·9	4·1	3·4; 5·0	< 0·001
SHEIA15 without vegetables and fruit	2·9	2·5; 3·3	3·3	2·7; 4·0	< 0·001	3·4	2·9; 3·8	4·2	3·5; 5·1	< 0·001
SHEIA15 without fibre	2·9	2·5; 3·3	3·1	2·6; 3·9	< 0·001	3·4	3·0; 3·9	4·1	3·4; 4·9	< 0·001
SHEIA15 without wholegrains	2·9	2·6; 3·4	3·2	2·7; 3·9	< 0·001	3·4	3·0; 3·9	4·2	3·4; 5·0	< 0·001
SHEIA15 without seafood	2·8	2·4; 3·2	3·4	2·8; 4·1	< 0·001	3·3	2·8; 3·7	4·4	3·7; 5·2	< 0·001
SHEIA15 without PUFA	2·9	2·5; 3·3	3·3	2·7; 4·0	< 0·001	3·4	2·9; 3·8	4·2	3·5; 5·1	< 0·001
SHEIA15 without MUFA	2·9	2·5; 3·3	3·3	2·7; 4·0	< 0·001	3·4	2·9; 3·9	4·2	3·5; 5·0	< 0·001
SHEIA15 without SFA	3·0	2·6; 3·4	3·1	2·6; 3·9	< 0·001	3·4	3·0; 3·9	4·1	3·4; 4·9	< 0·001
SHEIA15 without added sugar	2·9	2·5; 3·3	3·2	2·7; 4·0	< 0·001	3·4	2·9; 3·9	4·2	3·5; 5·0	< 0·001
SHEIA15 without red and processed meat	3·0	2·6; 3·6	3·0	2·6; 3·5	< 0·001	3·6	3·1; 4·3	3·7	3·1; 4·3	0·073

Abbreviations: GHGEs, greenhouse gas emissions; CO_2_e, carbon dioxide equivalents.

* The women and men were participants in the population-based Västerbotten Intervention Programme during the period 1990–2016. Values are medians and 1st; 3rd quartiles. Diet quality is estimated by the Swedish Healthy Eating Index for Adults (SHEIA15), which is based on the adherence to the Swedish dietary guidelines from 2015 and includes nine components. The SHEIA15 score and dietary GHGEs have been energy adjusted to 2000 kcal for women and 2500 kcal for men. Ranking into quintiles was adjusted by age groups (35–44 years, 45–54 years and 55–65 years). Quintile 1 is the lowest quintile, and Quintile 5 is the highest quintile. The dietary GHGEs include emissions from farm to industry gate.

† Differences between quintile one and quintile 5 were tested using the non-parametric Mann–Whitney U test.

## Discussion

We had two aims with the current study. First, we aimed to assess the association between the proposed diet quality index SHEIA15, which was developed to estimate adherence to the 2015 Swedish FBDGs, and all-cause mortality, thus assessing the index’ ability to predict health outcomes. Second, we aimed to investigate the differences in dietary GHGEs of participants with increasing adherence to the 2015 Swedish FBDGs, according to SHEIA15, to learn if a higher adherence to the FBDGs is associated with lower dietary GHGEs. Importantly, we found a consistent trend of lower all-cause mortality HR for both women and men with higher SHEIA15 scores. We also found a consistent trend of lower estimated dietary GHGEs among women and men with higher SHEIA15 scores.

These consistent trends of lower all-cause mortality HR with increasing SHEIA15 scores suggest that SHEIA15 is a suitable index to assess diet quality among Swedish adults. Adherence to the 2015 Swedish FBDGs and association with all-cause mortality were simultaneously captured, suggesting that following the 2015 Swedish FBDGs is related to longevity. SHEIA15 has previously been proposed as an index to identify healthy dietary patterns and as a tool to capture overall diet quality among Swedish adolescents^(9)^. The previous study by Moraeus et al. indicated that a higher SHEIA15 score was associated with significantly higher intakes of positive diet components, such as vegetables, wholegrains, fish, fibre and PUFA, and significantly lower intakes of negative diet components, such as added sugar, SFA and red and processed meat^(9)^. Other diet quality indices assessing adherence to Swedish FBDGs have been developed previously and the indices’ associations with health outcomes have been assessed. In 2011, a diet quality index based on the 2005 Swedish Nutrition Recommendations and Swedish dietary guidelines called DQI-SNR was developed by Drake et al.^(23)^. The DQI-SNR is based on the six components SFA, PUFA, seafood, dietary fibre, fruit and vegetables and sucrose^(23)^. A higher index score was shown to be inversely associated with all-cause mortality and incidence of cardiovascular events among men, and with incidence of cardiovascular events among women, within the population-based Malmö Diet and Cancer cohort^(24,25)^. In 2022, a diet quality index further building on the DQI-SNR but based on five components of the 2015 Swedish FBDGs (dietary fibre, fish, fruit and vegetables, added sugar and red and processed meat), called the Swedish Dietary Guidelines Score (SDGS), was shown to be inversely associated with stroke HR within the Malmö Diet and Cancer cohort^(26)^. Hence, adherence to Swedish FBDGs seems to be related to positive health outcomes. Nevertheless, only the endpoint all-cause mortality was included in the Cox analyses in the current study, and associations with endpoints that are specifically dietary-related could thus be further assessed to ensure that SHEIA15 is a suitable indicator for healthy dietary patterns.

Increasing SHEIA15 scores were further associated with consistent trends of lower dietary GHGEs among both women and men in the current study, indicating that a higher adherence to the 2015 Swedish FBDGs is beneficial not only for longevity but also for climate sustainability. Similar results to ours were found in a population-based Dutch cohort study from 2017, investigating the associations between adherence to the 2015 Dutch dietary guidelines, estimated by the Dutch Healthy Diet index 2015 (DHD15-index), and dietary GHGEs, dietary land use and also all-cause mortality HR^(27)^. A higher adherence to the 2015 Dutch dietary guidelines was found to be associated with lower dietary GHGEs, less land use and also lower mortality HR among both women and men^(27)^. Nevertheless, sensitivity analyses in the current study suggested that the indicated association between higher SHEIA15 score and lower dietary GHGEs was mostly driven by the component red and processed meat, since indicated differences in dietary GHGEs between quintiles one and five of SHEIA15 score were neutralised when this component was excluded. This result is not surprising since the dietary-related GHGEs are considerably higher for the food group red and processed meat compared with all other food groups^(28)^, and it also indicates the importance of the dietary guideline to decrease the consumption of red and processed meat in reducing dietary GHGEs. Still, GHGEs are only one environmental consequence of food production and consumption. The 2015 Swedish FBDGs are developed to encompass several environmental impacts^(29)^, and our analyses of associations between SHEIA15 and dietary GHGEs need to be repeated also with other environmental impacts to ensure that there are no detrimental trade-offs. Since FBDGs are directed towards the general population, other important aspects to facilitate following the guidelines include affordability and acceptability.

The primary strength of the current study is that the data used come from the large population-based cohort VIP, encompassing reliable data on all-cause mortality as well as standardised and validated methods of assessments of dietary intake and other lifestyle factors. Limitations of the current study include that solely baseline dietary data were used in the analyses, and eventual dietary or lifestyle changes during the follow-up time have not been considered. Further, misreporting is a problem with all self-reported dietary data, and even though energy adjustments were performed to partly adjust for this, indicated diet-health associations could have been attenuated. The range in SHEIA15 scores was narrow in our sample, especially among women. However, many composite scores and indexes share this property of having a narrow theoretical range. Even so, because SHEIA15 is constructed as the sum of several ratios, an unlimited number of values within the range are possible. Importantly, significant associations with all-cause mortality and dietary GHGEs were detected. However, it would be interesting to evaluate our identified associations in populations with a broader range of SHEIA15. Also, uncertainties in life cycle assessment GHGE data exist, impacting the estimation of dietary GHGEs of the study participants^(30)^. Furthermore, although potential confounders have been adjusted for, residual confounding effects are still possible. Lastly, the current study is an observational study and no causal relationship between dietary patterns and mortality can hence be confirmed.

### Conclusion

A higher adherence to Swedish food-based dietary guidelines, estimated by the diet quality index SHEIA15, was associated with lower all-cause mortality hazards as well as lower estimated dietary GHGEs among women and men in a Swedish population. SHEIA15 is thus suggested as a predictor of both all-cause mortality and dietary GHGEs, indicating that a higher adherence to dietary guidelines is beneficial for both longevity and climate sustainability.

## Supplementary material

For supplementary material accompanying this paper visit https://doi.org/10.1017/S1368980023001295. click here to view supplementary material
