# Supplementary material for: Adherence to the Swedish dietary guidelines and the impact on mortality and climate in a population-based cohort study
Source: Public Health Nutr. 2023 Jul 3;26(11):2333–42. doi: 10.1017/S1368980023001295 (PMC10641615; doi:10.1017/S1368980023001295)
Supplement: Supplementary file 1 [file S1368980023001295sup001.docx]

**Adherence to the Swedish dietary guidelines and the impact on mortality and climate in a population-based cohort study**

***Supplementary material***

*Anna Strid, Elinor Hallström, Anna Karin Lindroos, Bernt Lindahl, Ingegerd Johansson, Anna Winkvist**

*Department of Internal Medicine and Clinical Nutrition, The Sahlgrenska Academy, University of Gothenburg, Gothenburg, Sweden (A.S., AK.L., A.W.)*

*Department of Agriculture and Food, RISE – Research Institutes of Sweden, Lund, Sweden (E.H.)*

*Department of Risk and Benefit Assessment, Swedish Food Agency, Uppsala, Sweden (AK.L.)*

*Sustainable Health, Department of Public Health and Clinical Medicine, Umeå University, Umeå, Sweden (B.L., A.W.)*

*Department of Odontology, Umeå University, Umeå, Sweden (I.J.)*

**Corresponding author:* Anna Winkvist, Department of Internal Medicine and Clinical Nutrition, The Sahlgrenska Academy, University of Gothenburg, Box 459, SE-405 30, Gothenburg, Sweden. E-mail: [anna.winkvist@nutrition.gu.se](mailto:anna.winkvist@nutrition.gu.se)

**SUPPLEMENTARY TABLE 1.** Baseline characteristics of participating women (*n*=49,124) in the population-based prospective cohort Västerbotten Intervention Programme between 1990-2016, classified into quintiles according to their diet quality estimated by SHEIA15*

|  | | Quintile 5 (*n*=9824) | | Quintile 4 (*n*=9826) | | Quintile 3 (*n*=9825) | | | Quintile 2 (*n*=9826) | | | Quintile 1 (*n*=9823) | |
| --- | --- | --- | --- | --- | --- | --- | --- | --- | --- | --- | --- | --- | --- |
|  | | Mean or  *n* (%) | SD | Mean or  *n* (%) | SD | Mean or  *n* (%) | SD | | Mean or  *n* (%) | SD | | Mean or  *n* (%) | SD |
| Age† (y) | | 48.5 | 8.0 | 48.0 | 8.0 | 47.7 | 8.0 | | 47.5 | 8.0 | | 47.6 | 8.0 |
| Body mass index† (kg/m^2^) | | 25.6 | 4.7 | 25.6 | 4.6 | 25.5 | 4.4 | | 25.6 | 4.6 | | 25.8 | 4.9 |
|  | Underweight, <18.5, *n* (%) | 82 (0.8) | – | 107 (1.1) | – | 98 (1.0) | – | | 92 (0.9) | – | | 152 (1.5) | – |
|  | Normal, 18.5–25.0, *n* (%) | 5038 (51.3) | – | 4997 (50.9) | – | 5168 (52.6) | – | | 5212 (53.0) | – | | 4959 (50.5) | – |
|  | Overweight, >25.0–30.0, *n* (%) | 3118 (31.7) | – | 3195 (32.5) | – | 3166 (32.2) | – | | 3034 (30.9) | – | | 3058 (31.1) | – |
|  | Obese, >30.0, *n* (%) | 1586 (16.1) | – | 1527 (15.5) | – | 1393 (14.2) | – | | 1488 (15.1) | – | | 1654 (16.8) | – |
| Physical activity, *n* (%) | |  |  |  |  |  |  |  | |  |  | |  |
|  | Inactive | 1373 (14.0) | – | 1537 (15.7) | – | 1730 (17.7) | – | | 1862 (19.0) | – | | 2158 (22.1) | – |
|  | Moderately inactive | 2641 (27.0) | – | 2968 (30.3) | – | 3079 (31.5) | – | | 3139 (32.1) | – | | 3229 (33.1) | – |
|  | Moderately active | 2609 (26.7) | – | 2711 (27.7) | – | 2696 (27.6) | – | | 2837 (29.0) | – | | 2749 (28.1) | – |
|  | Active | 3159 (32.3) | – | 2566 (26.2) | – | 2280 (23.3) | – | | 1945 (19.9) | – | | 1632 (16.7) | – |
| Level of education, *n* (%) | |  |  |  |  |  |  |  | |  |  | |  |
|  | Basic level, 9 years | 2569 (26.3) | – | 3040 (31.2) | – | 3482 (35.7) | – | | 3976 (40.8) | – | | 4386 (45.0) | – |
|  | High school | 2725 (27.9) | – | 2783 (28.5) | – | 2801 (28.7) | – | | 2837 (29.1) | – | | 3100 (31.8) | – |
|  | University | 4478 (45.8) | – | 3932 (40.3) | – | 3463 (35.5) | – | | 2940 (30.1) | – | | 2259 (23.2) | – |
| Smoking, *n* (%) | |  |  |  |  |  |  |  | |  |  | |  |
|  | Currently smoking | 1311 (13.4) | – | 1573 (16.1) | – | 1913 (19.7) | – | | 2299 (23.6) | – | | 2997 (30.8) | – |
|  | Have smoked | 3199 (32.8) | – | 3026 (31.0) | – | 2792 (28.7) | – | | 2633 (27.1) | – | | 2476 (25.5) | – |
|  | Do not smoke | 5249 (53.8) | – | 5148 (52.8) | – | 5025 (51.6) | – | | 4795 (49.3) | – | | 4254 (43.7) | – |

*The women were participants in the population-based Västerbotten Intervention Programme during the period 1990–2016. Diet quality is estimated by the Swedish Healthy Eating Index for Adults (SHEIA15), which is based on the adherence to the Swedish dietary guidelines from 2015. Ranking into quintiles was adjusted by age groups (35–44 y, 45–54 y, and 55–65 y). Quintile 1 is the lowest quintile and Quintile 5 the highest quintile. The SHEIA15 score has been energy adjusted to 2000 kcal for women. †Adjusted for age and year of study participation.

**SUPPLEMENTARY TABLE 2.** Baseline characteristics of participating men (*n*=47,651) in the population-based prospective cohort Västerbotten Intervention Programme between 1990-2016, classified into quintiles according to their diet quality estimated by SHEIA15*

|  | | Quintile 5 (*n*=9529) | | Quintile 4 (*n*=9531) | | Quintile 3 (*n*=9531) | | | Quintile 2 (*n*=9531) | | | Quintile 1 (*n*=9529) | |
| --- | --- | --- | --- | --- | --- | --- | --- | --- | --- | --- | --- | --- | --- |
|  | | Mean or  *n* (%) | SD | Mean or  *n* (%) | SD | Mean or  *n* (%) | SD | | Mean or  *n* (%) | SD | | Mean or  *n* (%) | SD |
| Age† (y) | | 48.2 | 8.0 | 48.0 | 8.0 | 47.8 | 8.0 | | 47.8 | 8.0 | | 48.0 | 8.0 |
| Body mass index† (kg/m^2^) | | 26.3 | 3.7 | 26.5 | 3.7 | 26.6 | 3.7 | | 26.6 | 3.9 | | 26.8 | 4.0 |
|  | Underweight, <18.5, *n* (%) | 16 (0.2) | – | 25 (0.3) | – | 21 (0.2) | – | | 26 (0.3) | – | | 43 (0.5) | – |
|  | Normal, 18.5–25.0, *n* (%) | 3640 (38.2) | – | 3547 (37.2) | – | 3478 (36.5) | – | | 3473 (36.4) | – | | 3239 (34.0) | – |
|  | Overweight, >25.0–30.0, *n* (%) | 4508 (47.3) | – | 4574 (48.0) | – | 4608 (48.3) | – | | 4564 (47.9) | – | | 4589 (48.2) | – |
|  | Obese, >30.0, *n* (%) | 1365 (14.3) | – | 1385 (14.5) | – | 1424 (14.9) | – | | 1468 (15.4) | – | | 1658 (17.4) | – |
| Physical activity, *n* (%) | |  |  |  |  |  |  |  | |  |  | |  |
|  | Inactive | 1565 (16.5) | – | 1677 (17.7) | – | 1805 (19.0) | – | | 1794 (18.9) | – | | 1957 (20.6) | – |
|  | Moderately inactive | 2582 (27.2) | – | 2841 (29.9) | – | 2963 (31.2) | – | | 3082 (32.4) | – | | 2926 (30.8) | – |
|  | Moderately active | 2493 (26.2) | – | 2698 (28.4) | – | 2773 (29.2) | – | | 2808 (29.6) | – | | 2923 (30.8) | – |
|  | Active | 2865 (30.1) | – | 2284 (24.0) | – | 1960 (20.6) | – | | 1816 (19.1) | – | | 1679 (17.7) | – |
| Level of education, *n* (%) | |  |  |  |  |  |  |  | |  |  | |  |
|  | Basic level, 9 years | 2870 (30.3) | – | 3433 (36.2) | – | 3809 (40.2) | – | | 4260 (45.0) | – | | 4649 (49.1) | – |
|  | High school | 2967 (31.3) | – | 3186 (33.6) | – | 3280 (34.6) | – | | 3305 (34.9) | – | | 3382 (35.7) | – |
|  | University | 3647 (38.5) | – | 2856 (30.1) | – | 2392 (25.2) | – | | 1912 (20.2) | – | | 1434 (15.2) | – |
| Smoking, *n* (%) | |  |  |  |  |  |  |  | |  |  | |  |
|  | Currently smoking | 1107 (11.8) | – | 1373 (14.6) | – | 1759(18.7) | – | | 2054 (21.9) | – | | 2654 (28.4) | – |
|  | Have smoked | 2967 (31.5) | – | 3071 (32.7) | – | 3051 (32.5) | – | | 2940 (31.3) | – | | 2761 (29.5) | – |
|  | Do not smoke | 5341 (56.7) | – | 4949 (52.7) | – | 4577 (48.8) | – | | 4394 (46.8) | – | | 3942 (42.1) | – |

*The men were participants in the population-based Västerbotten Intervention Programme during the period 1990–2016. Diet quality is estimated by the Swedish Healthy Eating Index for Adults (SHEIA15), which is based on the adherence to the Swedish dietary guidelines from 2015. Ranking into quintiles was adjusted by age groups (35–44 y, 45–54 y, and 55–65 y). Quintile 1 is the lowest quintile and Quintile 5 the highest quintile. The SHEIA15 score has been energy adjusted to 2500 kcal for men. †Adjusted for age and year of study participation.

**SUPPLEMENTARY TABLE 3.** Energy-adjusted reported food intakes and estimated energy and nutrient intakes of participating women (*n*=49,124) in the population-based prospective cohort Västerbotten Intervention Programme between 1990-2016, classified into quintiles according to their diet quality estimated by SHEIA15*

|  | Quintile 5 (*n*=9824) | | Quintile 4 (*n*=9826) | | Quintile 3 (*n*=9825) | | Quintile 2 (*n*=9826) | | Quintile 1 (*n*=9823) | |
| --- | --- | --- | --- | --- | --- | --- | --- | --- | --- | --- |
|  | Median | 1^st^;3^rd^ quartiles | Median | 1^st^;3^rd^ quartiles | Median | 1^st^;3^rd^ quartiles | Median | 1^st^;3^rd^ quartiles | Median | 1^st^;3^rd^ quartiles |
| SHEIA15 score | 7.6 | 7.4;7.8 | 7.0 | 6.9;7.2 | 6.6 | 6.4;6.7 | 6.1 | 5.9;6.2 | 5.3 | 5.0;5.6 |
| Crude energy (g/day) | 1403 | 1146;1703 | 1430 | 1176;1732 | 1447 | 1195;1755 | 1454 | 1184;1774 | 1429 | 1142;1748 |
| Carbohydrates (E%) | 52 | 48;56 | 51 | 47;55 | 50 | 46;54 | 49 | 44;53 | 46 | 42;51 |
| Protein (E%) | 15 | 14;17 | 15 | 14;16 | 15 | 14;16 | 15 | 14;16 | 15 | 13;17 |
| Fat (E%) | 30 | 27;34 | 31 | 28;35 | 33 | 29;37 | 34 | 31;38 | 37 | 33;41 |
| PUFA (E%) | 6 | 5;7 | 5 | 4;6 | 5 | 4;6 | 5 | 4;6 | 4 | 4;5 |
| MUFA (E%) | 11 | 9;12 | 11 | 10;12 | 11 | 10;13 | 12 | 10;13 | 12 | 11;14 |
| SFA (E%) | 11 | 10;13 | 13 | 11;14 | 14 | 12;15 | 15 | 13;16 | 16 | 14;18 |
| Added sugars (E%) | 4 | 3;6 | 5 | 3;7 | 6 | 4;8 | 6 | 4;8 | 7 | 5;9 |
| Vegetables and fruits (g/day and 2000 kcal) | 584 | 448;778 | 450 | 328;632 | 345 | 250;485 | 264 | 189;360 | 175 | 113;260 |
| Fibre (g/day and 2000 kcal) | 29 | 26;33 | 26 | 23;30 | 24 | 21;27 | 21 | 18;24 | 17 | 15;20 |
| Wholegrains (g/day and 2000 kcal) | 56 | 43;70 | 45 | 32;58 | 37 | 24;49 | 28 | 17;41 | 17 | 10;28 |
| Fish (g/day and 2000 kcal) | 33 | 24;46 | 25 | 16;35 | 22 | 14;31 | 20 | 12;29 | 15 | 7;24 |
| Red and processed meat (g/day and 2000 kcal) | 58 | 43;73 | 61 | 45;78 | 63 | 46;83 | 66 | 49;90 | 75 | 52;107 |

Abbreviations: PUFA, polyunsaturated fatty acids; E%, percent of total energy; MUFA, monounsaturated fatty acids; SFA, saturated fatty acids.
*The women were participants in the population-based Västerbotten Intervention Programme during the period 1990–2016. Diet quality is estimated by the Swedish Healthy Eating Index for Adults (SHEIA15), which is based on the adherence to the Swedish dietary guidelines from 2015. This index ranges between 0 – 9. Ranking into quintiles was adjusted by age groups (35–44 y, 45–54 y, and 55–65 y). Quintile 1 is the lowest quintile and Quintile 5 the highest quintile. The SHEIA15 score and the food intakes have been energy adjusted to 2000 kcal. Food, energy, and nutrient intakes are estimated based on a food frequency questionnaire.

**SUPPLEMENTARY TABLE 4.** Energy-adjusted reported food intakes and estimated energy and nutrient intakes of participating men (*n*=47,651) in the population-based prospective cohort Västerbotten Intervention Programme between 1990-2016, classified into quintiles according to their diet quality estimated by SHEIA15*

|  | Quintile 5 (*n*=9529) | | Quintile 4 (*n*=9531) | | Quintile 3 (*n*=9531) | | Quintile 2 (*n*=9531) | | Quintile 1 (*n*=9529) | |
| --- | --- | --- | --- | --- | --- | --- | --- | --- | --- | --- |
|  | Median | 1^st^;3^rd^ quartiles | Median | 1^st^;3^rd^ quartiles | Median | 1^st^;3^rd^ quartiles | Median | 1^st^;3^rd^ quartiles | Median | 1^st^;3^rd^ quartiles |
| SHEIA15 score | 7.1 | 6.8;7.4 | 6.3 | 6.2;6.5 | 5.8 | 5.7;5.9 | 5.3 | 5.1;5.4 | 4.6 | 4.3;4.8 |
| Crude energy (g/day) | 1863 | 1516;2276 | 1908 | 1545;2330 | 1899 | 1540;2329 | 1891 | 1521;2328 | 1849 | 1461;2309 |
| Carbohydrates (E%) | 49 | 45;53 | 47 | 43;52 | 46 | 41;50 | 44 | 40;49 | 43 | 37;48 |
| Protein (E%) | 14 | 13;16 | 14 | 13;16 | 14 | 13;16 | 14 | 13;16 | 14 | 13;16 |
| Fat (E%) | 33 | 29;37 | 35 | 31;39 | 37 | 33;41 | 38 | 34;42 | 40 | 35;45 |
| PUFA (E%) | 6 | 5;8 | 6 | 5;7 | 5 | 4;7 | 5 | 4;6 | 5 | 4;6 |
| MUFA (E%) | 12 | 10;13 | 12 | 11;14 | 13 | 11;15 | 13 | 12;15 | 14 | 12;16 |
| SFA (E%) | 13 | 11;14 | 14 | 13;16 | 15 | 14;17 | 16 | 14;18 | 18 | 15;20 |
| Added sugars (E%) | 5 | 3;7 | 6 | 4;8 | 6 | 4;8 | 6 | 4;9 | 7 | 4;10 |
| Vegetables and fruits (g/day and 2500 kcal) | 372 | 262;514 | 246 | 169;339 | 189 | 128;267 | 147 | 94;212 | 98 | 58;155 |
| Fibre (g/day and 2500 kcal) | 30 | 26;35 | 26 | 22;30 | 23 | 20;27 | 21 | 18;24 | 17 | 14;21 |
| Wholegrains (g/day and 2500 kcal) | 63 | 46;80 | 45 | 29;61 | 33 | 20;47 | 23 | 14;37 | 15 | 10;25 |
| Fish (g/day and 2500 kcal) | 33 | 23;47 | 27 | 18;39 | 25 | 16;36 | 21 | 12;32 | 14 | 2;24 |
| Red and processed meat (g/day and 2500 kcal) | 70 | 53;87 | 78 | 59;101 | 85 | 63;110 | 92 | 66;123 | 112 | 79;146 |

Abbreviations: PUFA, polyunsaturated fatty acids; E%, percent of total energy; MUFA, monounsaturated fatty acids; SFA, saturated fatty acids.
*The men were participants in the population-based Västerbotten Intervention Programme during the period 1990–2016. Diet quality is estimated by the Swedish Healthy Eating Index for Adults (SHEIA15), which is based on the adherence to the Swedish dietary guidelines from 2015. This index ranges between 0 – 9. Ranking into quintiles was adjusted by age groups (35–44 y, 45–54 y, and 55–65 y). Quintile 1 is the lowest quintile and Quintile 5 the highest quintile. The SHEIA15 score and the food intakes have been energy adjusted to 2500 kcal. Food, energy, and nutrient intakes are estimated based on a food frequency questionnaire.
